# Hepatic progenitor cells express SerpinB3

**DOI:** 10.1186/1471-2121-15-5

**Published:** 2014-02-11

**Authors:** Gianmarco Villano, Cristian Turato, Santina Quarta, Mariagrazia Ruvoletto, Francesco Ciscato, Liliana Terrin, Rossella Semeraro, Claudia Paternostro, Maurizio Parola, Domenico Alvaro, Paolo Bernardi, Angelo Gatta, Patrizia Pontisso

**Affiliations:** 1 Department of Medicine-DIMED, University of Padua, Via Giustiniani 2, Padua 35128, Italy; 2 Division of Gastroenterology, Department of Scienze e Biotecnologie Medico-Chirurgiche, Fondazione Eleonora Lorillard Spencer Cenci, University Sapienza, Rome, Viale dell’Università 37, Rome 00185, Italy; 3 Department of Experimental Medicine and Oncology and Interuniversity Center for Liver Pathophysiology, University of Torino, Corso Raffaello 30, Torino 10125, Italy; 4 Department of Biomedical Sciences, University of Padua, Viale Colombo 3, Padua 35131, Italy

**Keywords:** Hepatic progenitor cells, C57BL/6J mouse, SerpinB3, Mouse model, LPS/D-Galactosamine

## Abstract

**Background:**

In the setting of liver injury hepatic progenitor cells are activated, counterbalancing the inhibited regenerative capacity of mature hepatocytes. Chronic activation of this compartment may give rise to a subset of liver tumours with poor prognosis. SerpinB3, a serpin over-expressed in injured liver and in primary liver cancer, has been shown to induce apoptosis resistance, epithelial to mesenchymal transition and to increase TGF-beta and Myc expression. Aim of the present study was to explore the presence of SerpinB3 in hepatic progenitor cells in human livers and in a mouse model of liver stem/progenitor cell activation.

Hepatic progenitor cells were analysed in foetal and adult livers at protein and transcriptional levels. To induce experimental activation of the liver stem/progenitor compartment, C57BL/6J mice were injected with lipopolysaccharide plus D-galactosamine and were sacrificed at different time points. Liver cDNA was amplified using specific primers for mouse-homologous SerpinB3 isoforms and automatically sequenced.

**Results:**

The presence of SerpinB3 in the progenitor cell compartment was detected in sorted human foetal and adult epithelial cell adhesion molecule (EpCAM) positive liver cells. By immunohistochemistry SerpinB3 was found in human cirrhotic livers in portal areas with progenitor cell activation showing ductular proliferation. CK-7, CK-19, EpCAM and CD-90 positive cell were also positive for SerpinB3. In the animal model, time course analysis in liver specimens revealed a progressive increase of SerpinB3 and a parallel decrease of activated caspase 3, which was barely detectable at 20 hours. Transcription analysis confirmed the presence of SerpinB3-homologous only in the liver of injured mice and sequence analysis proved its belonging to mouse Serpinb3b.

**Conclusion:**

SerpinB3 is highly expressed in hepatic stem/progenitor cell compartment of both foetal and adult livers.

## Background

The liver is characterized by a unique and remarkable capacity to regenerate upon various injuries, such as partial hepatectomy or toxic insults. Liver regeneration can usually be achieved by proliferation of the differentiated hepatocytes, without involvement of the stem/progenitor cell compartment. However, under severe and/or chronic liver damage conditions in which the proliferative capacity of normal adult hepatocytes is inhibited, the stem/progenitor cells contribute to the regeneration process [1]. This compartment, defined in rodents “oval cell compartment”, is composed by quiescent cells that originate from niches located in the canals of Hering and in normal bile ducts [2,3]. Oxidative stress conditions can induce activation of liver progenitor cells, known as ductular reaction in human livers, characterized by the expansion of the small biliary cells compartment, which can differentiate into biliary epithelial cells and hepatocytes [4]. The occurrence of progenitor cell proliferation in humans has been described in the late stages of cirrhosis and can give rise to a peculiar subset of primary liver tumors with poor prognosis [5]. SerpinB3 (SB3) is a member of the ovalbumin-serin protease inhibitor family (ov-serpins) [6] and within the normal liver its expression was seen in portal interlobular ducts, in the walls of hepatic arteries and sometimes in the endothelial cells of the portal vein, but not in normal adult hepatocytes, except some focal faint positivity of hepatocytes in the limiting plate [7]. However, chronically damaged hepatocytes express this serpin [8] and the highest levels are achieved in preneoplastic and neoplastic liver lesions [9-11]. *In vitro* studies have shown that SB3 protects neoplastic cells from apoptotic death induced by several kinds of stimuli [12] and the suggested molecular target location has been supposed upstream caspase 3 [13]. Recent data have revealed that SB3 increases the synthesis of Myc oncogene [7] and of transforming growth factor-beta (TGF-β) [8]. In addition, this serpin has been found to induce epithelial-to-mesenchymal transition, associated with β-catenin accumulation, increased cellular proliferation and invasiveness [14]. The squamous cell carcinoma antigen locus, which in humans encodes the nearly identical serpins SerpinB*3 (SCCA1)* and SerpinB*4 (SCCA2)*[15], in mouse encodes four full-length genes, *Serpinb3a*, *-b3b*, *-b3c*, and *-b3d,* indicating their relation to an ancestral serpin common to both sets of mammalian genes. This notion is supported by the phylogenetic relationship ascertained between the predicted amino acid sequences of the existing SCCA-related genes of human (SerpinB3 and -B4), mouse (*Serpinb3a*, *-3b*, *-3c*, and *-3d*) and rat (*Serpinb3a* and *-3b*) genome [16].

SB3 expression has not been studied so far in hepatic stem/progenitor cells and the effect of its possible presence in this compartment has not been investigated yet.

In this study we have explored the expression of SB3 in human hepatic stem/progenitor cells and in a mouse model of stem/progenitor cells induction, determined by the administration of lipopolysaccharide (LPS) and D-galactosamine (D-GalN) [17].

## Methods

### Immunohistochemistry in human liver cirrhosis

Liver specimens obtained from sequential liver sections of four patients with HCV-related cirrhosis (METAVIR F4) were immunostained for SB3, for cytokeratin 7 (CK7), cytokeratin 19 (CK19), thymocyte antigen 1 (Thy1 or CD90), tyrosine-protein kinase Kit (c-kit or CD117), transmembrane sialomucin protein (CD34) and epithelial cell adhesion molecule (EpCAM). The use of human liver bioptic material, obtained after written consent, conforms to the ethical guidelines of the 1975 Declaration of Helsinki and was approved by the Ethical Committee of the University of Torino. Immunohistochemistry was performed on sequential paraffin liver sections (2 micron thick) that were incubated with specific monoclonal antibodies (final dilutions 1:50) raised against SB3 (Santa Cruz Biothecnology Inc, Santa Cruz, CA, USA), CK-7 and CK-19 (Dako, Glostrup, Denmark), EpCAM (Miltenyi Biotec GmbH, Bergisch Gladbach, Germany), CD90, CD117 and CD34 (clones G7, 2B8 and MEC14.7, respectively; BioLegent, London, UK), used as liver progenitor cells markers [18-22]. Briefly, after microwave antigen retrieval, primary antibodies were labeled using EnVision, HRPlabelled System (Dako) antibodies directed against mouse antigens and visualized by 3’- diaminobenzidine substrate. Negative controls were performed by replacing the respective primary antibodies by isotype and concentrations matched irrelevant antibody [23].

### Human liver stem/progenitor cell isolation

Foetal livers (N = 4, weight 14–16 g) of 18 week gestational age were obtained by elective (trisomy 21) termination of pregnancy from the Department of Gynecology (Sapienza, University of Rome, Italy). Non tumoral adult liver specimens (3 different specimens) were obtained at the time of liver surgery for primary liver cancer in a patient with non-alcoholic steatohepatitis (NASH) from the Department of General Surgery and Organ Transplantation, University of Rome “La Sapienza”*.* The protocols for the use of foetal liver, obtained after written consent by elective (trisomy 21) termination of pregnancy and for adult liver, were approved by the Ethical Committee of Sapienza, University of Rome, Italy.

Human stem/progenitor cells were isolated according to Schmelzer E et al. [24,25]. Briefly, the liver was initially reduced in small fragments with lancets and then submitted to enzymatic digestion (30 min at 37°C) in a cell buffer containing 300 U/ml type I Collagenase (Sigma-Aldrich) and 0.3 mg/ml deoxyribonuclease (DNAse, Sigma-Aldrich). This resulted in a homogeneous cell suspension that was passed through pre-separation filters of 100 μ and enrichment for stem/progenitor cells was performed by magnetic immunoselection for epithelial cell adhesion molecule (EpCAM). For this purpose, magnetic microspheres conjugated with anti-EpCAM monoclonal antibody (Miltenyi Biotec GmbH, Bergisch Gladbach, Germany) were used. From the initial cell suspension (pre-sorting = 1.30×10^8^ cells), 1.5x10^7^ EpCAM + viable sorted cells were obtained. Isolated cells were submitted to flow Cytometric (FC) analyses as described [24,25]. Briefly, isolated cells were labeled with fluorescent primary antibodies (EpCAM-FITC, Miltenyi Biotec Inc., Bergisch Gladbach, Germany; NCAM-PE (neural cell adhesion molecule), R&D Systems Inc., Minneapolis, MN USA) or adequate isotype controls. Cells were analyzed by a FACScanto Flow Cytometer (Becton Dickson, Milan, Italy). Ten thousand events were acquired and analyzed by CellDiva software. Total RNA was extracted using the TRI REAGENTTM (Sigma-Aldrich, St. Louis, MO, USA), following the manufacturer’s instructions [26]. The isolated RNA was dissolved in 55 μL of RNase-free water and used for molecular analysis.

### Molecular techniques

#### Real time polymerase chain reaction in human liver cell preparations

Total RNA (up to 1 μg) from human liver cell preparations was reverse transcribed using Superscript II reverse transcriptase (Invitrogen, Carlsbad, CA, USA) and mRNA amplification for human SB3 was performed as previously described [27] using the following primers: sense SB3, 5′-GCAAATGCTCCAGAAGAAAG-3′; reverse SB3, 5′-CGAGGCAAAATGAAAAGATG-3′. The housekeeping gene Glyceraldehyde-3-phosphate dehydrogenase (GAPDH) (sense: 5′-TGGTAT TCGGGAAGGACTCATGAC-3′, reverse: 5′-ATGCCAGTGAGCTTCCCGTTCAGC-3′) was determined in parallel in all the amplification sets to assess the integrity of total RNA extracts.

#### Reverse-transcription polymerase chain reaction and cDNA sequencing in mouse livers

Polymerase chain reaction (PCR) analysis for SB3 was performed on total RNA extracted from frozen mouse liver samples. Total RNA (2.0 μg/sample) was used as a template to create complementary DNA [27] and quantified by spectrophotometry at 260 nm. To characterize the mouse-homologous serpin isoforms, the following set of conserved primers was used: sense: 5′- TTTTACACAAGTCCTTTGTGGAGG-3′, antisense: 5′-CTGGACACATGGAAGAGACACCAC-3′, which allows the amplification of the mouse Serpinb3a, -b3b and -b3c, homologous to human SerpinB3 and SerpinB4 [16,28]. The mRNA amplification reaction was performed as described previously [27]. The amplified product was then transcribed to cDNA and sequenced by direct sequencing, using an ABI 310 automated DNA sequencer (Apply Biosystems, Foster City, CA, USA), according to manufacturer instructions. All the obtained DNA sequencing data were already present in GenBank and their accession numbers were reported in the results section.

Homology search analysis to achieve maximal levels of identity of sequenced nucleotides was carried out by Basic Local Alignment Search Tool algorithm (BLAST), designed to explore all of the available mouse transcript sequence databases [29]. To determine the relatedness of the serpins from human and mouse, the deduced amino acid sequences were examined using the ClustalX multiple sequence alignment algorithm.

### Western blot analysis

The protein expression of mouse SB3, cleaved caspase 3, proliferative cell nuclear antigen (PCNA) and β-actin was assessed by Western blot analysis using cellular extracts of liver tissue, as described previously [27].

The expression of each protein was detected using the following primary antibodies: polyclonal anti-SB3 (Hepa-Ab, 1:462, kind gift of Dr. Fassina, Xeptagen, Venice, Italy), polyclonal anti-cleaved caspase 3 (1:1000, Cell Signaling Technology, Danvers, MA, USA), monoclonal anti-PCNA (1:100, Dako, Glostrup, Denmark), monoclonal anti-β actin (1:1000, Sigma-Aldrich). Anti-mouse IgG (1:1000, Amersham Bioscience, Arlington Height, IL, USA) and anti-rabbit IgG (1:2000, Sigma-Aldrich) were used as horseradish peroxidase conjugated secondary antibodies.

### Acute liver failure model

The study was carried out in adult C57BL/6J mice (Charles River Italia S.p.A, Calco, Lecco), aged 12–14 weeks with a weight range of 20–25 g, bred at the Animal Care Facility of the Experimental Surgery Division of the University of Padua.

To induce acute liver failure male C57BL/6J mice received in preliminary experiments increasing concentrations (3,3 -20 μg/Kg) of E. Coli lipopolysaccharide (LPS, Sigma-Aldrich, St. Louis, MO) plus 700 mg/Kg of D-galactosamine (D-GalN, Sigma-Aldrich) in PBS (1×) by intraperitoneal injection [30] and were sacrificed at different time points to assess the dose-dependent liver injury.

An additional group of 10 mice was inoculated with 5 μg/Kg of E. Coli LPS plus 700 mg/Kg of D-GalN, defined as the optimal dose to achieve 24 hours mortality. Within this group, a subgroup of 5 mice was sacrificed 5 hours after injection, while the remaining mice were sacrificed after 20 hours, by lethal dose of tiletamine hydrochloride. As control, a similar group of mice was inoculated with 200 μl of PBS (1×). Liver specimens were cut in two parts, one part was fixed with 4% paraformaldehyde in PBS, followed by paraffin embedding, while the other part was immediately frozen in liquid nitrogen and stored at -80°C for further analysis.

The experimental protocol was approved by the Animal Investigation Committee of the Italian Ministry of Health. All measures were taken to minimize any pain or discomfort for the animals.

### Statistical analysis

Statistical analysis was carried out using the Student’s t-test, the non parametric Mann–Whitney test and Wilcoxon matched pairs test, when appropriate; all tests were two-tailed. The significance was set as p < 0.05. All analyses were performed using GraphPad InStat software (San Diego, CA, USA).

## Results

### SerpinB3 in human liver progenitor cells

**Figure 1:**
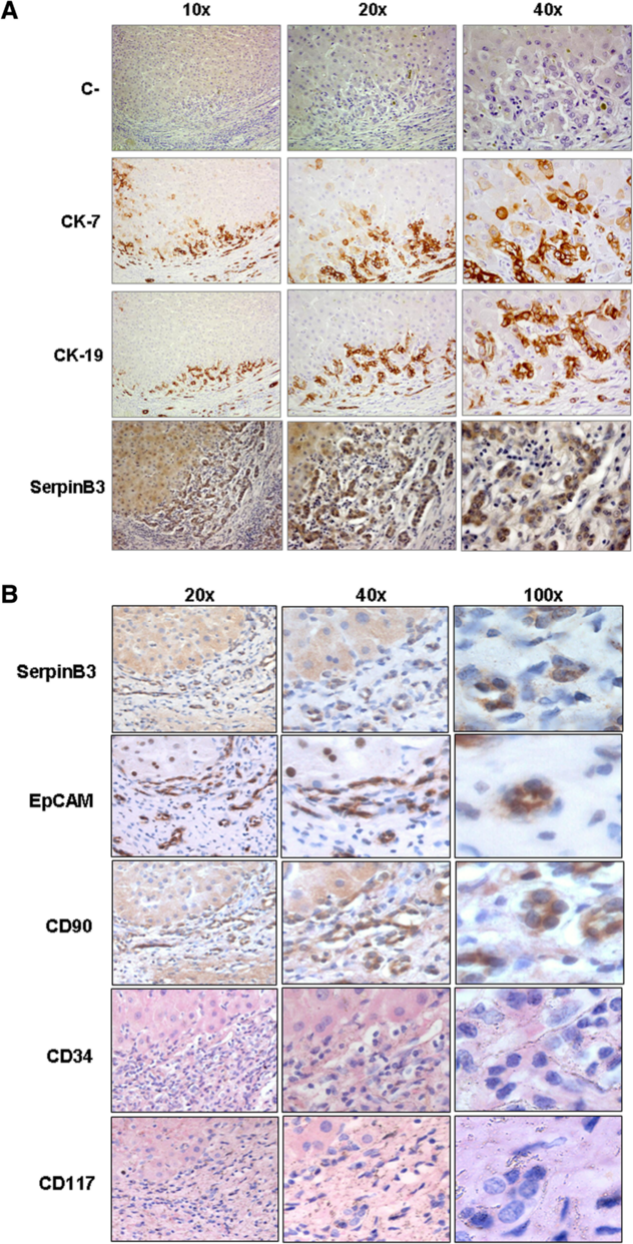
**Immunohistochemistry in human liver cirrhosis.** Immunohistochemistry on serial liver sections obtained from a cirrhotic patient with ductular proliferation. **A)** Representative images show negative control (C-) and immunostaining for cytokeratin 7 (CK-7), cytokeratin 19 (CK-19) and for SerpinB3. **B)** Serial liver sections immunostained for SerpinB3, EpCAM, CD-90, CD-34 and CD-117. Original magnification as indicated.

Immunohistochemistry was carried out in serial sections of human livers with HCV-related cirrhosis in order to evaluate the expression of SB3 in human progenitor cell compartment, typically found in portal tracts of cirrhotic livers as ductular reaction of CK-7 and CK-19 positive cells [4]. As shown in Figure 1A, staining of SB3 in consecutive liver sections clearly showed large numbers of CK-7 and CK-19 positive cells that also expressed SB3. In addition, similar areas positive for SB3 were also positive for EpCAM and CD-90, but negative for CD34 and for CD117 (Figure 1B), supporting the hypothesis that this serpin is expressed in this stem/progenitor cell compartment [18-22].

**Figure 2:**
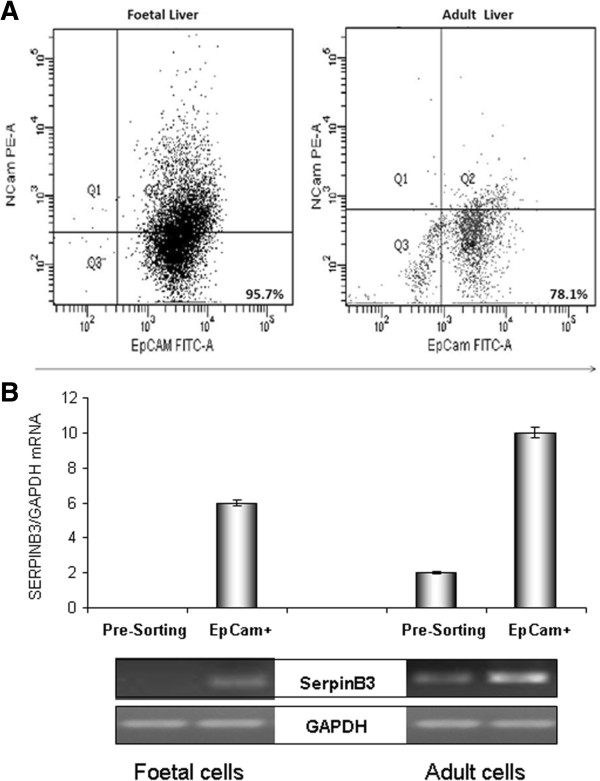
**Stem/progenitor cells analysis. A**: Flow cytometry analysis of stem/progenitor cells isolated from foetal or adult livers. Cells were isolated by magnetic immunoselection for EpCAM. Flow cytometry analysis, where cells were stained with anti-EpCAM and anti-NCAM antibodies, demonstrated the enrichment of EpCAM + cells. A significant number of EpCAM + cells were also positive for NCAM. **B**: RNA analysis for SerpinB3 in human liver stem/progenitor cells. In the real time amplification for human SerpinB3 data were normalized to GAPDH housekeeping gene and the y-axis represents the relative mean mRNA level of the SB3 gene expressed by 2^-ΔCt^^*^10^-5^, with the bars representing the standard error. In the lower panel agarose gel electrophoresis of individual corresponding samples is shown as example.

Stem/progenitor cells from foetal or adult livers were sorted for EpCAM by using magnetic beads. By flow cytometry analysis, after sorting for EpCAM, 79.0 ± 15.4% of freshly isolated cells were EpCAM positive for adult liver and 92 ± 5.2% for foetal liver (Figure 2A).

In keeping with immunohistochemistry, mRNA of SB3 was detectable in stem/progenitor cells sorted as EpCAM positive from human foetal and adult livers (Figure 2B).

### Animal model

C57BL/6J mice, treated with increasing concentrations of LPS (range: 3.3-20 μg/Kg) plus D-GalN displayed signs of acute liver failure with progressive changes of hepatic architecture and liver cell necrosis. The dose of 5 μg/Kg of LPS was defined as the optimal dose to carry on the experiments over a time frame of 5–20 hours.

#### Western blot analysis

**Figure 3:**
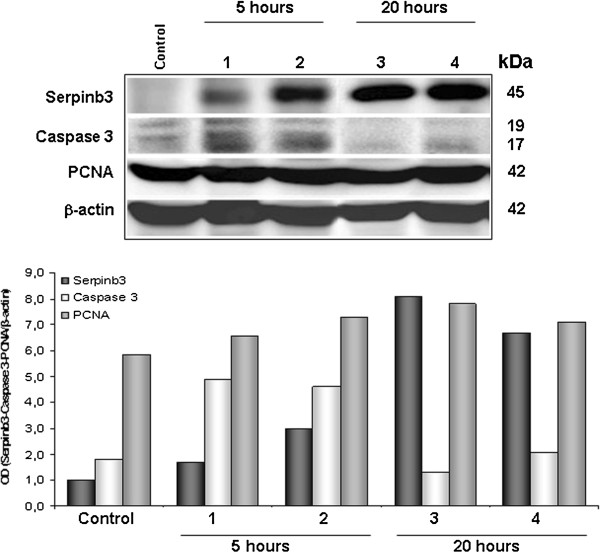
**Western blot analysis.** Upper Panel) Representative images of Western blot analysis for Serpinb3, activated Caspase 3 and PCNA in liver homogenates of two mice sacrificed at 5 hours (lanes 1,2) and of two mice sacrificed at 20 hours (lanes 3,4) after treatment with LPS/D-galactosamine. The liver of a control mouse has also been shown. Lower Panel) Densitometric analysis of the above results expressed as optical density (OD) of each molecule. The quantitative densitometric values are normalized to β-actin.

Western blot analysis of total liver lysates from control and LPS (5 μg/Kg)-DGalN injured mice was carried out at 5 hours and 20 hours after treatment. Densitometric examination showed that Serpinb3 protein became detectable 5 hours after liver injury, with a progressive increase over time. After 20 hours, the highest expression of Serpinb3 was associated with a marked decrease of the active form of caspase 3, supporting the parallel inhibition of apoptotic activity, while PCNA levels remained unchanged (Figure 3).

#### Sequence analysis of SerpinB3 homologues

**Figure 4:**
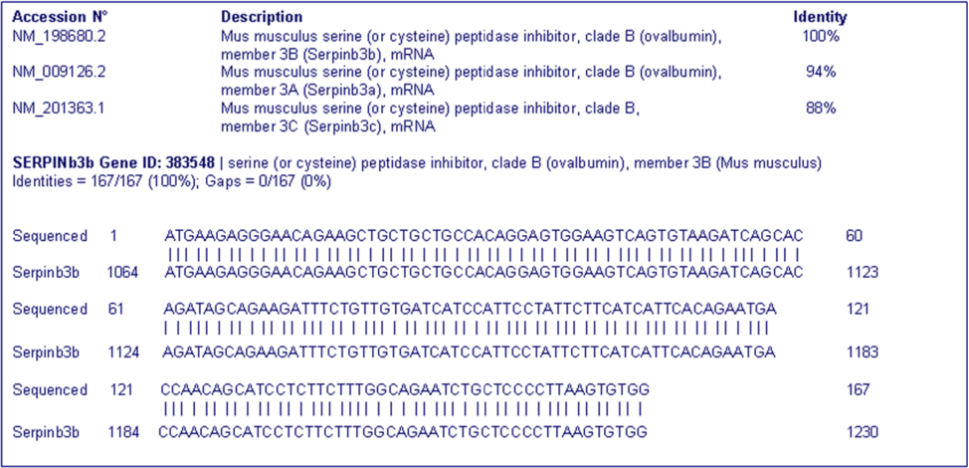
**Sequence analysis of Serpinb3b cDNA.** Sequence analysis of Serpinb3b cDNA confirmed the presence of SerpinB3-homologous only in the liver of injured mice, being not detectable in controls. Homology search analysis proved its belonging to mouse Serpinb3b.

To analyze the transcriptional products of the mouse SB3 isoforms, RT-PCR was carried out using primers for the conserved regions of the genomic isoforms already described in mouse [16,28]. In the liver of all treated mice, a single band at the expected size for mouse SB3 mRNA, corresponding to 214 bp, was detectable, while it was absent in controls. Direct sequencing of the amplified product was carried out and nucleotide sequence alignment proved its belonging to mouse Serpinb3b (GenBank accession number: NM_198680) (Figure 4), which has the highest sequence homology with human SerpinB3 (NM_006919). Furthermore, sequence homology analysis of Serpinb3b showed a 94% identity with Serpinb3a (NM_009126) and 88% with Serpinb3c (NM_201363).

## Discussion

Chronic human liver diseases are characterized by continuous liver damage and hepatocyte loss, with subsequent activation of the progenitor cell compartment [20]. Despite the capacity of the adult human and rodent liver to reply to loss of its cellular mass, it is widely acknowledged that impairment of the regenerative capacity of hepatocytes may result in proliferation and migration of cells forming ductular structures, initially close to the biliary tree. The capacity of these ductular structures to proliferate and differentiate into hepatocytes, in association with impaired liver regeneration, has led to the conclusion that they may represent progeny of facultative liver stem cells [31,32]. In recent years, considerable interest has been devoted to the hepatic progenitor cells, as tumors showing hepatic progenitor cell features have a worse prognosis and a higher recurrence rate compared to tumors lacking these characteristics [5,21].

The present study has provided evidence that in the human progenitor cell compartment SB3 is detectable in the same ductular structures that express stem/progenitor cell markers, with multiple differentiating capabilities [18,19,33]. Within the ductular reaction areas, a high proportion of CK-7 and CK-19 positive cells presented indeed a positive staining for SB3. This SB3 positive cell population was also positive for EpCAM and CD90, markers of mesenchymal and foetal liver stem cells. This is the first report, at the best of our knowledge, indicating that SB3 is present in human stem/progenitor cells of hepatic origin. The lack of the hematopoietic markers CD34 and CD117 in the same areas may exclude that this population derives from circulating hematopoietic cells, rather supporting its belonging to a distinct liver stem cell-like population [34,35].

In addition, to evaluate the expression of SB3 in the liver stem/progenitor cell compartment, EpCAM + cells were isolated by immunomagnetic sorting from foetal and adult human livers. A bulk of recent literature deals with the role of EpCAM + cells as hepatic resident stem/progenitor cells [36,37]. Moreover, microarray expression data from a panel of human tissue generated using GNF Expression Atlas 2 Data from U133A chip (publicy available through the UCSC Genome Browser website http://genome.ucsc.edu/), further confirms that EpCAM mRNA is robustly expressed in foetal liver. The detection of SB3 RNA in the EpCAM + cell fractions of human foetal and adult livers supports strongly the presence of this serpin in hepatic progenitor cells [22].

An animal model of stem/progenitor cell induction after acute liver injury determined by LPS/D-GalN [38] has also been studied. In this model hepatic injury is mediated by macrophages [39] and proinflammatory cytokines, including IL-1, IL-6, IL-12 and TNF-α [40] lead to hepatocyte necrosis and to massive hepatocyte apoptosis [41]. Members of the TNF family are the best characterized stimuli for stem/progenitor cell proliferation, an event that shares an unusual reciprocal relationship with the proliferation of hepatocytes [42]. In our experimental conditions, using total cell lysates, a progressive increase of mouse SB3 and a parallel decrease of activated caspase 3 was detectable in the early phase of acute liver injury. We cannot exclude the possibility that the observed increase in mouse SB3 may be contributed by other cell types, rather than the progenitors, since activated B lymphocytes have been also reported to express SB3 [43]. Transcription analysis confirmed the presence of SB3-homologous only in the liver of injured mice and sequence analysis proved its belonging to mouse Serpinb3b. This serpin, one of the four isoforms of the Serpinb3 mouse gene, is the counterpart of the human SB3 and shares 73% aminoacid similarity. Both are dual cross-class inhibitors of cysteine and serine proteinases [16,44], supporting the hypothesis that they retained these activities from an ancestral SCCA-like gene.

SB3 is a multifunctional protein that, beyond its antiprotease activity, makes cells more resistant to several killing mechanisms by inhibition of apoptosis [12]. The results obtained in LPS/D-GalN treated mice confirmed that overexpression of Serpinb3b in the liver was associated with a marked decrease of the activated caspase 3, which represents a well-defined hallmark of apoptosis.

SB3 has been detected recently in hepatoblastoma, the embryonal tumor of the liver, and a direct correlation was observed between its gene expression, the up-regulation of Myc oncogene and tumor extension [6]. In addition, *in vitro* experiments documented the primary involvement of SB3 in the up-regulation of Myc transcription [7]. This serpin has been also shown to be able to increase TGF-β expression in hepatic cell lines and in primary hepatocytes [8]. Chronic stimulation of hepatic progenitor cells by TGF-β has been shown recently to induce their transformation into cancer stem cell/tumor-initiating cells [45], suggesting the involvement of triggering autocrine and paracrine mechanisms in hepatocarcinogenesis. These observations, together with the fact that SB3 was found to promote epithelial to mesenchymal transition and to increase cell proliferation and invasiveness [14], may suggest a carcinogenic potential of hepatic progenitor cells expressing this serpin.

## Conclusion

SB3 was detectable in human progenitor cells compartment within the same ductular structures that express stem/progenitor cell markers, such as CK-7, CK-19, EpCAM and CD90. In addition, SB3 was detected in EpCAM + cell fractions sorted from human foetal and adult livers. The mouse homologous, Serpinb3b, was induced in a mouse model of liver stem/progenitor cell activation.

## Abbreviations

CD90: Thymocyte antigen 1; CD117: Tyrosine-protein kinase Kit; CK-7: Cytokeratin 7; CK-19: Cytokeratin 19; D-GalN: D-galactosamine; EpCAM: Epithelial cell adhesion molecule; LPS: Lipopolysaccharide; SB3: SerpinB3; TGF-β: Transforming growth factor-beta.

## Competing interests

The authors declare that they have no competing interests.

## Authors’ contributions

GV designed and performed the experiments, analyzed data and drafted the manuscript; CT, SQ, MR, FC, RS performed the experiments and the techniques described in the manuscript; CP and LT performed histological analysis; DA; MP, PB and AG were involved in the discussion and manuscript editing; PP was involved in the experimental design of the study, in the analysis of the data and manuscript editing. All authors read and approved the final manuscript.
